# Neurological complications in pediatric patients with SARS-CoV-2 infection: a systematic review of the literature

**DOI:** 10.1186/s13052-021-01066-9

**Published:** 2021-06-02

**Authors:** L. Siracusa, A. Cascio, S. Giordano, A. A. Medaglia, G. A. Restivo, I. Pirrone, G. F. Saia, F. Collura, C. Colomba

**Affiliations:** 1 Pediatric Infectious Diseases Unit, “G. Di Cristina” Hospital, ARNAS Civico, Via dei Benedettini 1, 90134 Palermo, Italy; 2 Department of Health Promotion, Maternal and Infant Care, Internal Medicine and Medical Specialties, University of Palermo, Palermo, Italy

## Abstract

**Objectives:**

To describe clinical characteristics, laboratory tests, radiological data and outcome of pediatric cases with SARS-CoV-2 infection complicated by neurological involvement.

**Study design:**

A computerized search was conducted using PubMed. An article was considered eligible if it reported data on pediatric patient(s) with neurological involvement related to SARS-CoV-2 infection. We also described a case of an acute disseminated encephalomyelitis (ADEM) in a 5-year-old girl with SARS-CoV-2 infection: this case was also included in the systematic review.

**Results:**

Forty-four articles reporting 59 cases of neurological manifestations in pediatric patients were included in our review. Most (32/59) cases occurred in the course of a multisystem inflammatory syndrome in children (MIS-C). Neurological disorders secondary to cerebrovascular involvement were reported in 10 cases: 4 children with an ischemic stroke, 3 with intracerebral hemorrhage, 1 with a cerebral sinus venous thrombosis, 1 with a subarachnoid hemorrhage, 1 with multiple diffuse microhemorrhages. Reversible splenial lesions were recognized in 9 cases, benign intracranial hypertension in 4 patients, meningoencephalitis in 4 cases, autoimmune encephalitis in 1 girl, cranial nerves impairment in 2 patients and transverse myelitis in 1 case. Five cases had Guillain-Barré syndrome (GBS) and two, including ours, had ADEM. Radiological investigations were performed in almost all cases (45/60): the most recurrent radiological finding was a signal change in the splenium of the corpus callosum. The presence of SARS-CoV-2 viral nucleic acid in the cerebrospinal fluid was proved only in 2 cases. The outcome was favorable in almost all, except in 5 cases.

**Conclusions:**

Our research highlights the large range of neurological manifestations and their presumed pathogenic pathways associated with SARS-CoV-2 infection in children. Nervous system involvement could be isolated, developing during COVID-19 or after its recovery, or arise in the context of a MIS-C. The most reported neurological manifestations are cerebrovascular accidents, reversible splenial lesions, GBS, benign intracranial hypertension, meningoencephalitis; ADEM is also a possible complication, as we observed in our patient. Further studies are required to investigate all the neurological complications of SARS-CoV-2 infection and their underlying pathogenic mechanism.

## Introduction

At the end of December 2019, many cases of atypical pneumonia of unknown origin were described in the city of Wuhan, China. In January 2020 a novel coronavirus, later called severe acute respiratory syndrome coronavirus 2 (SARS-CoV-2), was identified as the responsible of a new disease called coronavirus disease 2019 (COVID-19), declared pandemic by the World Health Organization (WHO) in March 2020.

As regards pediatric COVID-19 cases, unlike the clinical presentation of adult patients, a systematic review showed that the most commonly reported symptoms are fever, cough, pharyngitis and rhinorrhea; other frequent symptoms are headache, myalgia, rash, conjunctivitis, syncopal episodes and gastrointestinal manifestations such as vomiting, diarrhea, abdominal pain and difficulty in feeding [1–3].

In later April 2020, a novel syndrome in children and adolescents, termed multisystem inflammatory syndrome in children (MIS-C), related to SARS-CoV-2 infection was first described: initial reports surfaced in the United Kingdom and Italy [4, 5]. This condition, similar to Kawasaki disease and toxic shock syndrome, is characterized by persistent fever, a multisystem (≥ 2) organ involvement, elevation of inflammatory markers, link to SARS-CoV-2 (verified by polymerase chain reaction, serology or COVID-19 contact) and the exclusion of alternative diagnosis [6].

Regarding neurological involvement in COVID-19, severe neurological manifestations (encephalopathy, meningoencephalitis, stroke, seizure, Guillain-Barré syndrome, acute disseminated encephalomyelitis) have been reported mainly in adults [7, 8], while a few cases have been described in children. Two mechanisms were proposed to explain how SARS-CoV-2 may induce neurological damage: direct viral infection of nervous system through ACE2 receptors and inflammatory injury mediated by cytokines release [9]; in the latter case, neurological manifestations may be part of a MIS-C [10].

We describe here a case of acute disseminate encephalomyelitis (ADEM) related to SARS-CoV-2 infection in a pediatric patient and, with the aim of focus our attention on neurological manifestations of pediatric patients with SARS-CoV-2 infection, we performed a systematic review of the literature contextualizing our new case among all the cases retrieved in our search.

## Case report

A 5-year-old girl presented with a 3-day history of fever, neck swelling and erythematous skin rash. In the previous days an antigen rapid swab test for SARS-CoV-2 was performed with a negative result and she was treated with antibiotic and anti-inflammatory therapy.

On physical examination, the child was febrile (body temperature 39 °C); the skin was characterized by a maculopapular and not itchy rash on the face, neck, trunk and extremities, with palmoplantar involvement. A right laterocervical and painful lymphadenopathy, eyelid, hand and foot edema, red and fissuring lips and injected pharynx were present. The abdomen was painful and she complained of diarrhea. Cardiovascular, respiratory and neurological examinations were normal. Vital signs showed oxygen saturation 99%, heart rate 104 bpm, blood pressure 104/60 mmHg.

Blood tests revealed microcytic and hypochromic anemia, leukocytosis with lymphopenia, C-reactive protein (CRP) 20.55 mg/dL (normal value < 0.6), procalcitonin 4.5 ng/mL (normal value < 0.5), fibrinogen 649 mg/dL (normal range 200–400), D-dimer 2653 ng/mL (normal range < 500), ferritin 603 ng/mL (normal range 11–306), hyponatremia and hypoalbuminemia. Chest radiograph and abdomen ultrasound showed no abnormalities, while neck ultrasound revealed different oval-shape nodes with maximum diameter of 1.6 cm. Echocardiogram and electrocardiogram, performed to rule out Kawasaki disease, did not show pathological findings.

**Fig. 1:**
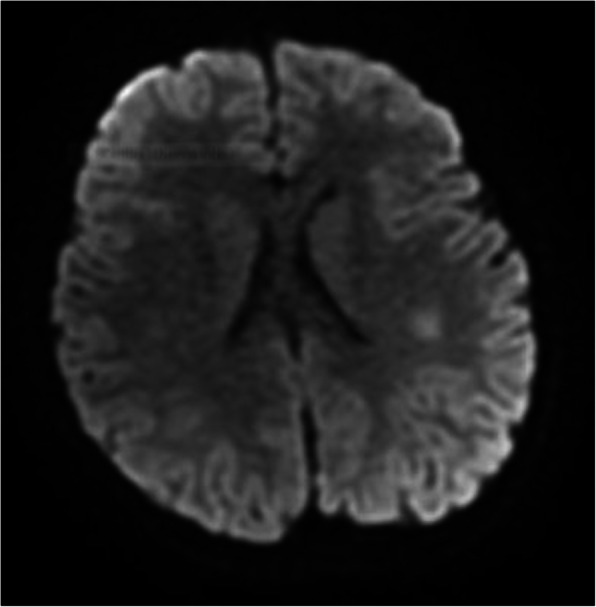
MRI DWI: lesion in the left subcortical white matter

**Fig. 2:**
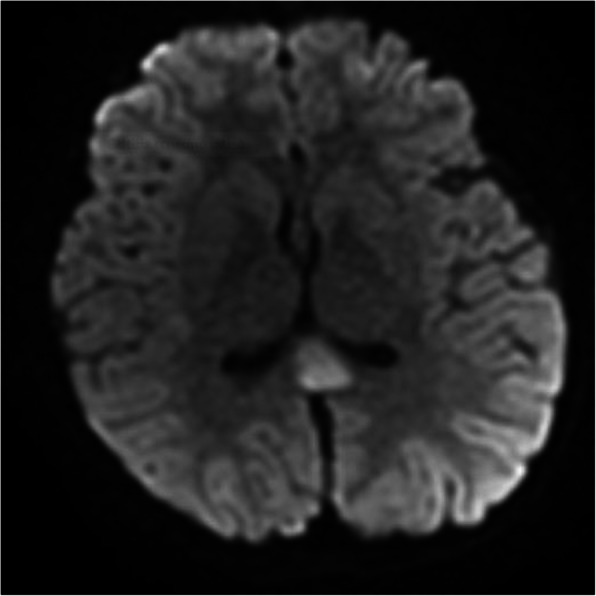
MRI DWI: lesion of the splenium of corpus callosum (transversal section)

**Fig. 3:**
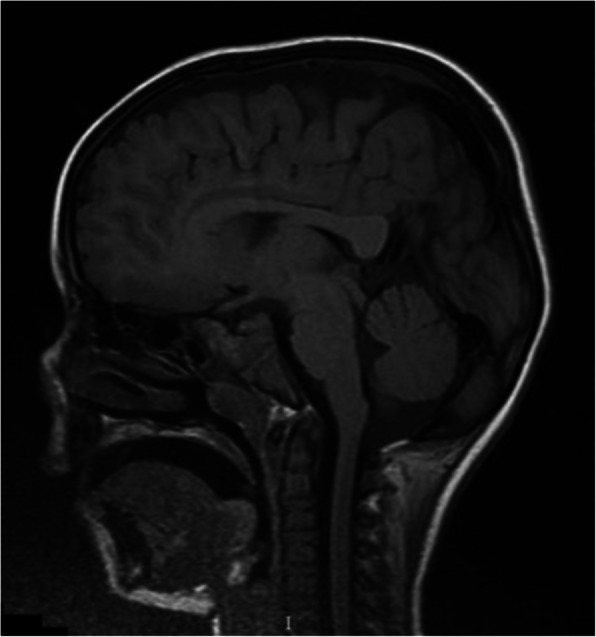
MRI DWI: lesion of the splenium of corpus callosum (sagittal section)

Two days after hospital admission, the girl became irritable; neck stiffness, muscular weakness and right Babinski sign were also found. In suspicion of viral encephalitis, she was treated with intravenous (IV) acyclovir 10 mg/kg three times a day. Brain MRI showed two lesions, one in the splenium of the corpus callosum and the other in the subcortical white matter of the left parietal lobe, that exhibit restricted diffusion without contrast enhancement (Figs. 1, 2 and 3).

Electroencephalogram (EEG) disclosed a generalized slowing of background activity. Cerebrospinal fluid (CSF) was tested: samples were acellular, with normal levels of proteins and glucose and no evidence of viral or bacterial infection (*Escherichia coli*, *Streptococcus pneumoniae*, *Haemophilus influenzae*, *Klebsiella pneumoniae*, *Streptococcus agalactiae*, *Neisseria meningitidis*, *Lysteria monocytogenes*, *Adenovirus*, *Herpes simplex virus 1–2*, *Varicella Zoster virus*, *Citomegalovirus*, *Epstein-Barr virus*, *Enterovirus*) on real-time polymerase chain reaction (RT-PCR). Tests for oligoclonal bands in CSF and serum neuronal autoantibodies (anti-NMDA, anti-VGCK, anti-AMPA) had negative results.

The molecular nasopharyngeal swab test for SARS-CoV-2 detected initially low viral load, while the second specimen was negative. A COVID-19 serology test, performed a week after the hospital admission, revealed IgG positive and IgM within grey-zone limits.

According to multi-organ involvement, neuroradiological findings, laboratory exams with elevated inflammatory parameters, temporal relationship with SARS-CoV-2 infection and exclusion of other causes, a diagnosis of ADEM in a patient with MIS-C was made; she started methylprednisolone 1 mg/kg/day IV and immunoglobulin 0.4 g/kg/day for 5 days IV, with a progressive resolution of the systemic hyperinflammatory state and improvement of neurological symptoms. Brain MRI, performed two weeks after the first one, demonstrated no abnormalities.

## Literature search

A computerized search was performed using PubMed, combining the terms (neurolog* OR CNS OR nervous OR encephal*) AND (COVID OR SARS-CoV-2 OR coronavirus) AND (baby OR child* OR pediatr*) with English language filter, to identify studies on neurological manifestations in children with SARS-CoV-2 infection, published until December 31, 2020. Furthermore, references within the included articles were scanned for other relevant papers. The following data were evaluated for each case: age, sex, comorbidities, clinical features, radiological and other neurological investigations, laboratory test for confirmation of SARS-CoV-2 infection and outcome; we also assessed if neurological complication occurred in the course of a MIS-C. We excluded articles that reported only aggregate data and that revealed the presence of coinfection with other microbes. The selected articles were reviewed by two independent authors and judged on their relevant contribution to the subject of the study. The Preferred Reporting Items for Systematic Review and Meta-Analysis (PRISMA) guidelines were followed [11].

## Results

**Fig. 4:**
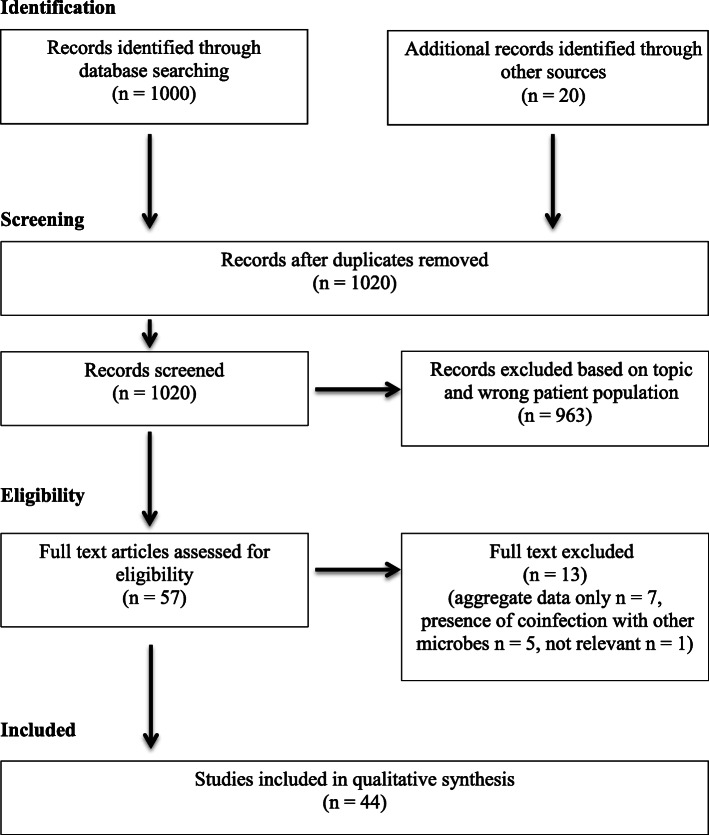
PRISMA study flow diagram: flow diagram of study identification, screening, eligibility, and included studies

After an extensive search in PubMed, 1000 articles were identified, along with 20 additional records detected though hand-searching (Fig. 4). 1020 records were screened; 963 were excluded after title and abstract screening and 13 were excluded after full-text review. We selected 44 studies for inclusion [5, 12–54], reporting 59 cases of neurological manifestations in pediatric patients with SARS-CoV-2 infection. Most of the articles were single case reports, 10 were case series. Clinical and radiological features, diagnosis and outcome of 60 patients (including our new case) are systematically reported in Table 1.

**Table 1:** Reported cases of neurological involvement during SARS-CoV-2 infection in children

Author/Country [Ref.]	Age/sex	Pre-existing medical conditions	Neurological symptoms	Respiratory symptoms	Other symptoms	Diagnosis of MIS-C	NP/CSF/Serology SARS-CoV-2	Radiology and other neurological investigations	Outcome
Abdel-Mannan et al./UK [12] 4 cases	8 y/M	No	Headache, meningism, confusion, muscular weakness	No	Fever, rash, abdominal pain, emesis, shock	Yes	Pos/Neg/ND	**CT:** hypodensity of the splenium of the corpus callosum	Improved
	9 y/M	No	Headache, confusion, ataxia, dysarthria, muscular weakness	No	Fever, rash, emesis, shock	Yes	Pos/Neg/ND	**MRI:** signal changes of the genu and splenium of corpus callosum and bilateral centrum semiovale with restricted diffusion	Recovered
	15 y/F	No	Confusion, dysarthria, dysphagia, muscular weakness	Yes	Fever, rash, emesis, shock	Yes	Pos/ND/Pos	**MRI:** signal changes in the splenium of corpus callosum and bilateral centrum semiovale with restricted diffusion	Improved
	15 y/F	No	Headache, confusion, muscular weakness	Yes	Fever, rash, emesis, shock	Yes	Pos/ND/Pos	**MRI:** signal change in the splenium of corpus callosum with restricted diffusion	Recovered
Abel et al./USA [13]	3 y/M	No	Irritability, hypotonia, muscular weakness	Yes	Fever, rash, emesis	Yes	Neg/Neg/Pos	**MRI:** restricted diffusion in the bilateral lateral thalamic nuclei **EEG:** moderate slow background activity	Improved, under physiotherapy
Asif et al./UK [14]	18 y/M	No	Headache, photophobia	No	Fever, cough and myalgia before neurological manifestations	No	Neg/ND/ND (previous diagnosis of COVID-19)	**CT venogram:** filling defects in the sigmoid and transverse sinuses bilaterally and in the straight and superior sagittal sinuses	Improved
Baccarella et al./US A[15]2 cases	9 y/M	No	Headache, diplopia, right abducens nerve palsy	No	Fever, abdominal pain	Yes	Neg/Neg/Pos	**MRI:** normal **LP:** elevated opening pressure	Recovered
	6 y/M	No	Headache, diplopia, right abducens nerve palsy	No	NR	Yes	Pos/Neg/Pos	**MRI:** finding consistent with elevated intracranial pressure	Recovered
Basirjafari et al./Iran [16]	9 y/M	No	Headache, bilateral fixed mydriasis	Yes	Fever, abdominal pain	No	Pos/ND/ND	**CT:** hyperdensity at basal cisterns, interhemispheric and bilateralSylvian fissures suggesting of subarachnoid hemorrhage and reduction of white matter density (brain edema)	Died
Bektas et al./Turke y[17]2 cases	10 y/M	No	Visual hallucinations, personality changes	Yes	Fever, diarrhea, rash, hands and feet edema	Yes	Neg/ND/Pos	**MRI:** hyperintensity in the splenium of corpus callosum with restricted diffusion **EEG:** slowed background activity	Recovered
	11 y/F	No	Personality changes	Yes	Fever, diarrhea, rash, conjunctivitis, hypotension	Yes	Neg/ND/Pos	**MRI:** hyperintensity in the splenium of corpus callosum with restricted diffusion **EEG:** slowed background activity	Recovered
Bhatta et al./USA [18]	11 y/M	No	Seizure	No	No	No	Pos/NR/ND	**CT:** normal	Recovered
Burr et al./USA [19]	23 m/F	No	Irritability, hyperkinetic movements of head, arms and legs	No	Fever	No	Pos/Neg/Pos	**MRI:** normal **NMDAR-IgG** positivity	Recovered
Chiotos et al./US A[20]4 cases	14 y/F	No	Headache	Yes	Fever, rash, diarrhea	Yes	Neg/ND/Pos	ND	Recovered
	12 y/M	No	Altered mental status, irritability	Yes	Fever, fissured lips, abdominal pain, diarrhea, shock	Yes	Neg/ND/Pos	ND	Recovered
	5 y/F	No	Altered mental status, irritability, nuchal rigidity	No	Fever, conjunctivitis, shock	Yes	Neg/ND/Pos	ND	Recovered
	5 y/F	No	Irritability, nuchal rigidity	No	Fever, rash, conjunctivitis, fissured lips, swollen hands, emesis, diarrhea, shock	Yes	Pos/ND/Pos	**CT:** diffuse cerebral edema	Recovered
Curtis et al./India [21]	8 y/M	No	Muscolar weakness, paralysis and paresthesia of the lower limbs	No	No	No	Pos/Neg/Pos	**MRI:** enhancement of the posterior nerve roots from T11 to cauda equine **LP:** albuminocytologic dissociation	Improved
de Miranda Henriques-Souza et al./Brazil [22]	12 y/F	No	Headache, muscular weakness, tetraplegia	Yes	Fever, rash	Yes	Pos/Neg/ND	**MRI:** bilateral and symmetric areas of restricted diffusion involving the subcortical and deep white matter. Extensive cervical myelopathy	Improved
De Paulis et al./Brazil [23]	4 y/F	No	Confusion, lethargy	Yes	Fever, emesis, rash, palpebrae, hands and feet edema, cracked lips, shock	Yes	Neg/Neg/Pos	**CT:** normal **LP:** pleiocytosis and elevated protein	Improved
Emami et al./Iran [24]	2.9 y/M	Allergy to cow milk	Seizure, altered mental status, dysarthria	No	Fever	No	Pos/ND/NR	**MRI:** right occipital mass andintracerebral hemorrhage **EEG:** generalized slowing (pathology of the mass: normal brain tissue with dilated vessels and haemorrhage)	Recovered
Enner et al./USA [25]	14 y/F	No	Seizure and central apnea	Yes	Fever, nasal congestion, myalgia	No	Pos/Neg/ND	**MRI:** normal **EEG:** epileptiform abnormalities	Improved
Frank et al./Brazil [26]	15 y/M	No	Ascending weakness froma the lower to the upper limbs, headache	No	Fever	No	Pos/Neg/Pos	**MRI:** normal **Electroneurography:** acute motor axonal neuropathy	Improved, under physiotherapy
Gaur et al./ U K[27]2 cases	12 y/M	NR	Headache, lethargy	No	Fever, diarrhea, conjunctivitis, shock	Yes	Neg/ND/Pos	**MRI:** hyperintensity in the splenium of corpus callosum with restricted diffusion	Recovered
	9 y/M	NR	Lethargy, ataxia, dysarthria	No	Fever	No	Neg/ND/NDPos broncho-alveolar lavage	**MRI:** hyperintensity in the splenium of corpus callosum and in the deep cerebral white matter with restricted diffusion	Recovered
Gulko et al./USA [28]	13 y/F	No	Headache, muscular weakness, speech difficulty	No	No	No	Pos/ND/ND	**CT:** left frontal hypodensity concerning for ischemic infarct. **MRI:** hyperintensity with restricted diffusion in the left frontal, parietal and temporal lobes; stenosis of the left middle cerebral artery	Improved
Kaur et al./Mexico [29]	3 y/F	No	Quadriparesis and paresthesia	Yes	Neurogenic respiratory failure	No	Pos/Neg/ND	**MRI:** swelling of the cervical spinal cord involving most of the transverse aspect of the spinal cord, extending from the lower medulla to the midthoracic level	Quadriparesis
Khalifa et al./Saudi Arabia [30]	11 y/M	No	Muscular weakness, hypotonia, paresthesia in the lower limbs	Yes	Fever and cough before neurological manifestations	No	Pos/NR/ND	**MRI:** cauda equina nerve root enhancement **LP:** albuminocytologic dissociation	Recovered
Kim et al./USA [31]	7 y/M	No	Headache, emesis	No	Fever, abdominal pain	Yes	Pos/Neg/ND	**CT:** diffuse cerebral edema **EEG:** generalized voltage attenuation	Died
Lin et al./USA [32]	13 y/F	No	Dizziness, gait instability, auditory hallucinations	Yes	Fever, diarrhea, emesis, hypotension	Yes	Pos/Neg/Pos	**MRI:** hyperintensity in the splenium of corpus callosum with restricted diffusion **EEG:** slow background activity	Recovered
Lorenz et al./Germany [33]	40 w/F	No	Lethargy, hyperexcitable	Yes	Fever	No	Pos/Neg/ND	**US:** normal	Recovered
Manji et al./Tanzania [34]	12 y/M	No	Progressive paresis, bilateral facial nerve paresis	Yes	Fever and cough before neurological manifestations	No	Pos/ND/ND	ND	Died
McAbee et al./ USA [35]	11 y/M	No	Seizure	No	Fever	No	Pos/Neg/ND	**CT:** normal **EEG:** intermittent frontal delta activity **LP:** pleiocytosis	Recovered
Mirzaee et al./Iran [36]	12 y/M	No	Seizure, dysarthria, hemiparesis	No	No	No	Pos/Pos/ND	**MRI:** acute infarction with narrowing of the left middle cerebral artery	Improved, under rehabilitation
Moreno-Galarraga et al./Spain [37]	2 m/F	No	Headache, seizure	No	Diarrhea Flu-like symptoms before neurological manifestations	No	Pos/NR/ND	**MRI:** normal **LP:** normal	Recovered
Natarajan et al./India [38]	13 y/F	No	Headache, irritability, seizure	No	Fever	No	Pos/Neg/ND	**MRI:** normal **LP:** pleiocytosis	Recovered
Paybast et al./Iran [39]	14 y/F	NR	Progressive paresthesia, muscular weakness, headache, dizziness	No	Flu-like symptoms before neurological manifestations	No	Pos/ND/ND	**LP:** albuminocytologic dissociation	Improved
Raj et al./India [40]	2 y/M	No	Seizure	No	Fever, diarrhea, hypotension	Yes	Pos/Neg/Neg	ND	Recovered
	15 m/M	No	Seizure	No	Fever, rash, conjunctivitis, cheilitis	Yes	NR/ND/NR(COVID-19 contact)	ND	Recovered
	8 m/M	NR	Seizure	No	Fever	No	Pos/ND/ND	ND	Recovered
Regev et al./Israel [41]	16 y/M	No	Headache, nuchal rigidity	No	Fever, abdominal pain, rash, conjunctivitis, pharyngitis, shock	Yes	Pos/ND/Pos	**MRI:** multiple low attenuating small lesionsin the subcortical white matter, internal and external capsule and in the anterior and posterior part of the corpus callosum, suggesting microhemorrhages	Recovered
Roussel et al./France [42]	6 y/F	Sickle cell disease, cerebral vasculopathy, HSCT	Impairment of V-VII-IX cranial nerves	Yes	No	No	Pos/Neg/ND	**MRI:** cranial nerves enhancement (left hypoglossal nerve and bilateral facial nerves)	Improved
Saeed et al./Iran [43]	3 y/M	No	Seizure	No	Fever, hypotension	Yes	Pos/Neg/ND	**CT:** cerebral edema **MRI:** intracerebral hemorrhage in the right occipital lobe	Recovered
Savić et al./Kuwait [44]	13 y/F	No	Altered mental status, right side weakness	No	No	No	Pos/ND/ND	**CT:** left side frontoparietal intracerebral hematoma with intraventricular extension **CT angiography:** pseudoaneurysm of the frontoparietal branch of the left middle cerebral artery	Not improved
Schupper et al./USA [45]	5 y/M	No	Right mydriasis	Yes	Fever, abdominal pain, shock	Yes	NR/NR/Pos	**CT:** a right middle cerebral artery infarction, cerebral edema and diffuse contralateral subarachnoid hemorrhage	Died
Seth et al./India [46]	15 y/M	NR	Headache, emesis, photophobia	No	Fever before neurological manifestations	No	Pos/Neg/ND	**MRI:** normal **LP:** elevated opening pressure and pleiocytosis	Recovered
Shenker et al./USA [47]	12 y/M	NR	Seizure	No	Fever, rash, conjunctivitis, neck swelling, cracked lips, hypotension	Yes	Pos/Neg/ND	**MRI:** normal **EEG:** focal epilepsy arising in the central region	Recovered
Swarz et al./USA [48]	9 y/M	No	Seizure	No	Fever, emesis	No	Pos/ND/ND	**MRI:** normal **EEG:** delta activity in the right hemisphere	Recovered
Theophanous et al./USA [49]	6 y/M	Prematurity, chromosome 17 and 19 deletions, submucosal palate cleft, atrial and ventricular septal defects, agammaglobulinemia with hyper-IgM, hypospadias, asthma, OSAS, gastrostomy	Right facial nerve palsy	No	No	No	Pos/ND/ND	ND	Recovered
Tiwari et al./India [50]	9 y/F	No	Headache, right hemiplegia, right facial nerve palsy	Yes	Fever, conjunctivitis, emesis	Yes	Pos/Neg/Pos	**CT:** multifocal hypodensities in the genu and body of corpus callosum, left basal ganglia and bilateral thalami suggestive of infarcts **CT angiography:** multifocal stenosis of both intracranial internal carotid arteries, right middle cerebral artery, both A2 segments of the anterior cerebral arteries and M2/M3segments of both middle cerebral arteries	Improved, under rehabilitation
Verdoni et al./Ital y[5]5 cases	7 y/M	No	Meningism	Yes	Fever, conjunctivitis, changes in lips and oral cavity, diarrhea	Yes	Pos/ND/Pos	ND	Recovered
	7.7 y/F	Congenital adrenal hyperplasia	Meningism	No	Fever, conjunctivitis, changes in lips and oral cavity, diarrhea	Yes	Neg/ND/Pos	ND	Recovered
	5 y/M	No	Meningism	No	Fever, rash, conjunctivitis hands and feet anomalies	Yes	Neg/ND/Pos	ND	Recovered
	5.5 y/M	No	Meningism	No	Fever, rash, conjunctivitis hands and feet anomalies	Yes	Neg/ND/Pos	ND	Recovered
	5.5 y/M	No	Drowsiness	Yes	Fever, rash, conjunctivitis hands and feet anomalies, diarrhea	Yes	Neg/ND/Pos	ND	Recovered
Verkuil et al./USA [51]	14 y/F	No	Headache, right abducens nerve palsy	Yes	Fever, diarrhea, rash, shock	Yes	Neg/ND/Pos	**MRI:** finding consistent with elevated intracranial pressure **LP:** elevated opening pressure	Recovered
Vivanti et al./France^a^ [52]	3 d/M	Prematurity	Irritability, opisthotonos	No	Feeding difficulty	No	Pos/Neg/ND	**MRI:** hyperintensity of the periventricular and subcortical frontal and parietal white matter	Improved
Yousefi et al./Iran [53]	9 y/F	NR	Headache, diplopia, photophobia, meningism	No	Fever	No	Neg/Pos/ND	**LP:** pleiocytosis, elevated protein, decreased glucose	Recovered
Zombori et al./UK [54]	17 y/F	Cornelia de Lange syndrome	Seizure	Yes	Fever	Yes	Pos/ND/ND	**MRI:** multifocal cortical, cerebellar and thalamic swelling areas **EEG:** bilateral independent periodic lateralized epileptiform discharges	Improved, under rehabilitation
Our case	5 y/F	No	Irritability, nuchal rigidity	No	Fever, rash, diarrhea, neck swelling	Yes	Pos/ND/Pos	**MRI:** two lesions, one in the splenium of the corpus callosum and the other in the subcortical white matter of the left parietal lobe, with restricted diffusion	Recovered

**Abbreviations:** *y* years; *m* months; *w* weeks; *d* days; *F* female; *M* male; *NP* nasopharyngeal; *CSF* cerebrospinal fluid; *MRI* magnetic resonance imaging; *CT* computerized tomography; *US* ultrasound; *EEG* electroencephalogram; *LP* lumbar puncture; *Pos* positive; *Neg* negative; *NR* not reported; *ND* not done; *HSCT* hematopoietic stem-cell transplantation; *OSAS* obstructive sleep apnea syndrome

^a^transplacental transmission of SARS-CoV-2 infection

There were 35 boys and 25 girls. The median age was 9 years. All children had no comorbidity, except 7 patients with no reported data and 6 patients with underlying conditions: a 3-year-old male with allergy to cow milk [24], a 6-year-old girl with sickle cell disease, complicated by cerebral vasculopathy, who underwent hematopoietic stem cell transplantation [42], a 6-year-old male with history of prematurity, chromosome 17 and 19 deletions, submucosal cleft palate, atrial and ventricular septal defects, immune deficit, hypospadias, asthma, obstructive sleep apnea syndrome and gastrostomy [49], a female with congenital adrenal hyperplasia [5], a male born preterm [52] and a 17-year-old female with Cornelia de Lange syndrome [54]. Four children were under 1 year old: one case of transplacental transmission of SARS-CoV-2 was demonstrated in a neonate born to a mother infected in the last trimester [52].

As regards neurological symptoms, the most commonly reported were headache in 2/3 of cases, altered mental status (from irritability and confusion to lethargy) in 32% of cases, seizure in 14/60 patients, muscular weakness in 14/60 children and meningism in 10/60.

Concerning neurological manifestations, we recognized acute cerebrovascular accidents in 10 children (4 cases of ischemic stroke, 3 cases of intracerebral hemorrhage, a subarchnoid hemorrhage, a case of multiple diffuse microhemorrhages, a cerebral sinus venous thrombosis), reversible splenial lesions in 9 cases, GBS in 5 persons, benign intracranial hypertension or pseudotumor cerebri in 4 patients, meningoencephalitis in 4 cases, autoimmune encephalitis in 1 girl, ADEM in 2 children (including ours), cranial nerves impairment in 2 patients and transverse myelitis in 1 case. Furthermore we found one report of severe encephalopathy with bilateral thalamic lesions and one article of fatal cerebral edema.

Fever was recorded in 75% of cases, while respiratory symptoms were present in 23/60 children. Six patients had flu-like symptoms before the onset of neurological complications. More than half of patients (55%) showed neurological complications in the course of a MIS-C, associated with a multisystem organ involvement (especially mucocutaneous, gastrointestinal and cardiac).

Radiological investigations (CT, MRI and/or ultrasound) were performed in almost all cases (45/60): the most recurrent radiological finding was a signal change in the splenium of the corpus callosum (12/60).

The diagnosis of SARS-CoV-2 infection was made according to the presence of SARS-CoV-2 viral nucleic acid in the nasopharyngeal swab in 29 cases and positive serology in 15 children; both nasopharyngeal swab and serology were positive in 11 patients. The presence of SARS-CoV-2 viral nucleic acid in the CSF was proved only in 2 cases (associated with a positive nasopharyngeal swab in 1 case). The outcome was favorable in almost all cases; 5 children died.

## Discussion

We described a case of ADEM in a pediatric patient with MIS-C related to SARS-CoV-2 infection. The diagnosis of ADEM was established according to the consensus criteria of the International Pediatric Multiple Sclerosis Study Group in 2013: a polyfocal, clinical central nervous system (CNS) event with a presumed inflammatory demyelinating cause; an encephalopathy that cannot be explained by fever; no new clinical and MRI findings emerging 3 months or more after the onset; abnormal brain MRI during the acute phase [55]. The close temporal relationship between encephalopathy and SARS-CoV-2 infection in our patient allowed us to consider the novel coronavirus as the trigger of the immune-mediated response against CNS, as already reported for other human coronavirus [56]. Furthermore our patient fulfilled the criteria for the diagnosis of MIS-C: she presented fever, mucocutaneous involvement, lymphadenopathy, diarrhea and neurological symptoms associated with elevated inflammatory markers and the presence of antibodies against SARS-CoV-2; unfortunately, the search for the novel coronavirus in the CSF was not performed, because a validated test was not available.

As recommended by American College of Rheumatology (ACR) [6], the first-tier agents for MIS-C treatment are IV immunoglobulin (typically 1–2 g/kg) and/or low to high doses of glucocorticoids (from 1 to 2 mg/kg/day to a bolus of 20–30 mg/kg/day for 3 days); acute treatment approach for pediatric ADEM is high-dose IV glucocorticoids for 3 or 5 days (either 10–30 mg/kg/day methylprednisolone or 1 mg/kg/day dexamethasone) followed by an oral steroid tapering or IV immunoglobulin at a total dose of 1–2 g/kg, administered either as a single dose or divided in 5 days (usually 400 mg/kg/day) [57]. Our girl was treated with glucocorticoids and immunoglobulin with a complete recovery; the outcome was favorable.

Afterwards, we have conducted a systematic review of the neurological complications during SARS-CoV-2 infection in pediatrics. Headache, irritability, drowsiness and seizure are the most frequent symptoms, that could be signs of different neurological conditions or neuroimaging abnormal findings: ischemic stroke, cerebral hemorrhage, benign intracranial hypertension, encephalitis, GBS, ADEM, splenial lesions. Furthermore, we observed that neurological investigations, especially radiological examinations, were not performed in all patients, especially in those with mild symptoms; in these cases, it is not clear what neurological condition is associated to SARS-CoV-2 infection.

The clinical observations summarized above suggest that SARS-CoV-2 could be responsible for many neurological manifestations, which can be divided into three different scenarios, related to the presumed pathophysiologic mechanism:

1. Neurological involvement during COVID-19;
2. Neurological involvement that arises after the recovery from COVID-19;
3. Neurological involvement during MIS-C.

The first condition could be caused by direct invasion of CNS by the virus through hematogenous dissemination or neuronal retrograde dissemination. In hematogenous dissemination, the virus can pass to the bloodstream and then enters the brain by either infecting endothelial cells of the blood-brain barrier or epithelial cells of the blood-CSF barrier in the choroid plexus, though the binding between spike protein and ACE2 receptor; furthermore, coronavirus can infect leukocytes, that disseminate towards other tissues and cross the blood-brain barrier to access the CNS (the so-called Trojan horse mechanism) [58]. In neuronal retrograde dissemination, the virus can gain access to CNS though the infection of olfactory neurons, using retrograde axonal transport [58]. This pathophysiologic mechanism could explain how SARS-CoV-2 can induce encephalitis and vasculitis leading to cerebrovascular accidents; the detection of the virus in the CSF samples using RT-PCR is an important sign of its neurotropism.

The second condition could be related to a post-infectious immune-mediated mechanism: SARS-CoV-2 might induce an autoimmune response after a latent period following the infection illness [59], correlated to the hypothesis of “molecular mimicry” between microbial and self-antigens. For example, GBS is characterized by ascending paralysis, occurring after the resolution of COVID-19 symptoms (fever and cough): it is caused by a cross-reaction against gangliosid-components of the peripheral nerves [60].

The third condition, the most recurrent observed in this review, could be explained though indirect mechanism caused by the novel coronavirus: the cytokine storm, characterized by high levels of tumor necrosis factor-alpha (TNF-α), interleukin (IL)-1β, IL-6, IL-12, and interferon gamma (INFγ) [59]. The integrity of the blood-brain barrier may be disrupted by cytokine-driven injury without CNS direct invasion by the virus [59]. Moreover, the hyperinflammatory state can lead to a pro-coagulable state: initial vasculitis causes the disruption of vascular integrity, the exposure of thrombogenic basement membrane and, finally, the activation of the clotting cascade [9]. Children with MIS-C exhibit alteration of inflammatory biomarkers (procalcitonin, CRP, fibrinogen, ferritin, D-dimer, IL-6), that suggest a possible involvement of the immune system in the pathogenesis of this syndrome [6]. Many observational studies about clinical characteristics of patients with MIS-C have reported the presence of neurological involvement: children could complain of headache, confusion, altered mental status, stiff neck or meningism [10, 61–65]. In the course of MIS-C, neurological complications, such as ADEM (our case), pseudotumor cerebri [15, 46, 51], cerebral edema [20, 31], seizure [40, 47], cerebral stroke [45, 50] and cytotoxic lesions of the corpus callosum [13, 17, 27, 32] have been described and included in this review. During hyperinflammatory state, the corpus callosum, especially the splenium, is highly vulnerable to excess of cytokines and glutamate release from astrocytes because of its high concentration of cytokines and glutamate receptors: this higher density leads to a tendency of cytotoxic edema of the corpus callosum when cytokine storm occurs [66]. Despite the great variability of neurological manifestations, from mild to severe ones, the prognosis is favorable in the majority of cases.

This systematic review has several limitations due to the quality of the selected studies (all articles are case reports or case series and do not represent the full population) and the potential impact of publication bias.

## Conclusions

Our research highlights the large range of neurological manifestations and their presumed pathogenic pathways associated with SARS-CoV-2 infection in children. CNS involvement could be isolated, developing during COVID-19 or after its recovery, or arise in the course of a MIS-C. The most reported neurological manifestations are cerebrovascular accidents, reversible splenial lesions, GBS, benign intracranial hypertension, encephalitis, cranial nerves impairment, transverse myelitis; ADEM is also a possible complication, as we observed in our patient. Outcome is good in almost all cases. Further studies are required to investigate all the neurological complications of SARS-CoV-2 infection and their underlying pathogenic mechanism.

## Data Availability

The datasets used and/or analyzed during the current study are available from the corresponding author on reasonable request.
